# Unveiling the Potential of Liquid Biopsy in HER2-Positive Breast Cancer Management

**DOI:** 10.3390/cancers14030587

**Published:** 2022-01-24

**Authors:** Ana Godoy-Ortiz, Alfonso Alba-Bernal, Javier Pascual, Iñaki Comino-Méndez, Emilio Alba

**Affiliations:** 1 Unidad de Gestión Clínica Intercentros de Oncología Médica, Hospitales Universitarios Regional y Virgen de la Victoria, 29010 Malaga, Spain; anagodort@gmail.com (A.G.-O.); alfonsoalba@uma.es (A.A.-B.); javier.pascual@ibima.eu (J.P.); 2 The Biomedical Research Institute of Málaga (IBIMA-CIMES-UMA), 29010 Malaga, Spain; 3 Centro de Investigación Biomédica en Red de Cáncer (CIBERONC), 28029 Madrid, Spain

**Keywords:** HER2-positive breast cancer, early breast cancer, metastatic breast cancer, liquid biopsy, circulating-tumour DNA, circulating-tumour cells

## Abstract

**Simple Summary:**

Breast cancer (BC) is the most prevailing cancer in women worldwide. Amongst the different BC subtypes, human epidermal growth factor receptor 2 (HER2)-positive tumours are characterised by an overexpression of the HER2 membrane receptor. Nowadays, HER2-status assessment relies on immunohistochemical methodologies in the tumour tissue, which could be complemented by novel methodologies to improve the clinical management of these patients. In this regard, liquid biopsy is an easy, rapid, and minimally invasive tool to obtain circulating tumour components from body fluids. Herein, by reviewing the published studies, we aim to decipher the clinical validity of liquid biopsy in both early and metastatic HER2-positive BC.

**Abstract:**

Invasive breast cancer (BC) is the most common cancer in women with a slightly increasing yearly incidence. BC immunohistochemical characterisation is a crucial tool to define the intrinsic nature of each tumour and personalise BC patients’ clinical management. In this regard, the characterisation of human epidermal growth factor receptor 2 (HER2) status guides physicians to treat with therapies tailored to this membrane receptor. Standardly, a tumour solid biopsy is therefore required, which is an invasive procedure and has difficulties to provide the complete molecular picture of the tumour. To complement these standard-of-care approaches, liquid biopsy is a validated methodology to obtain circulating tumour components such as circulating tumour DNA (ctDNA) and circulating tumour cells (CTCs) from body fluids in an easy-to-perform minimal-invasive manner. However, its clinical validity in cancer is still to be demonstrated. This review focusses on the utilisation of both ctDNA and CTCs in early and metastatic HER2-positive BC tumours. We discuss recently published studies deciphering the capacity of liquid biopsy to determine the response to neoadjuvant and adjuvant therapies as well as to predict patients’ outcomes.

## 1. Introduction

Breast cancer (BC) is the most common cancer among women worldwide and one of the major causes of cancer-related mortality in women [1]. With peak incidence between 35 and 75 years, most cases develop sporadically, and less than 10% of them are hereditary due to germline mutations [2]. The clinical management of BC has improved remarkably in the last decades by advancements in surgery and (neo)adjuvant therapies in early stages. These have made it possible to achieve a 5 year overall survival rate above 90% [3]. Nonetheless, BC can spread through distant metastases, which could worsen the 5-year survival rate to as low as 28% [4], and therapeutic strategies in these cases are mostly palliative in nature. The main obstacle for designing effective treatment approaches in BC is the complex heterogeneity of these tumours [5].

BC can be classified into four molecular subtypes according to the specific gene expression profiles of 50 genes or the so-called PAM50 assay: luminal A, luminal B, basal-like, and human epidermal growth factor receptor 2 (HER2)-enriched [6,7]. Among luminal tumours, the luminal A subtype has a higher expression of hormone-activated genes, low levels of proliferation markers and good survival rates, while Luminal B tumours have poorer prognosis, are characterised by higher histological grade, increased expression of tumour proliferation-associated genes and can display overexpression of HER2 [8]. On the other hand, the basal-like subtype presents low levels of luminal-related genes, low HER2 expression and high levels of proliferation genes being the intrinsic subtype with the poorest rates of survival [7].

As mentioned above, the HER2-enriched tumours are one of the subtypes defined by the PAM50 BC tumour subtyping and are characterised with the highest *ERBB2* gene expression amongst all subtypes both at RNA and protein level. Additionally, HER2-enriched tumours also show an increased expression of tumour proliferation-related genes [9,10]. The HER2 protein, encoded by the *ERBB2* gene, is a receptor belonging to the EGFR family [2]. Notably, the HER2 overexpression is not limited to this subtype as we can find HER2-positive tumours (overexpressing the HER2 receptor) with luminal A, luminal B, or even basal-like subtypes. Additionally, it is also possible to find HER2-enriched subtypes in histologically HER2-negative tumours, which can give rise to significant differences in response to treatment and biological evolution [7,11,12].

In clinical practice, HER2 positivity is determined by immunohistochemistry (IHC) and/or by in situ hybridisation (ISH) in order to tailor targeted therapeutic approaches [2]. The HER2 status assessment was originally standardised by The American Society of Clinical Oncology and the College of American Pathologists (ASCO/CAP) that published guidelines recommending to test HER2 protein overexpression by IHC and *ERBB2* gene amplification by ISH using FFPE tumour tissue. The ASCO/CAP guideline was updated in 2018, when the criteria were refined to systematise the testing algorithm for controversial HER2 categorisation. For these cases, it is required to have more strict interpretation for ISH together with a concomitant IHC [13]. Approximately 15–20% BC tumours have HER2 overexpression and/or amplification, and over 50% of these also coexpress hormone receptors [11].

The HER2 protein is a biomarker with a reported heterogeneity in BC [14]. In general, critical genomic changes in BC occur mostly during cancer progression, which can create significant variability between primary tumours and its metastases [5,15]. In addition, systemic treatments create dynamic molecular pressure, which can lead to more biological heterogeneity [16]. Ideally, the collection of serial tumour biopsies and an extensive longitudinal monitoring could capture the abovementioned heterogeneity and depict the disease changes caused by treatments and progression. HER2 status is a clear example of cancer heterogeneity [11] since it has been demonstrated that HER2 expression can change from primary disease to metastases by 9–60% [17,18,19,20]. This phenomenon can lead to an inappropriate treatment selection if HER2 status is not reassessed throughout different disease stages. Considering all the above, it is paramount to find new methodologies permitting a close disease monitoring and HER2 status evaluation to provide a proper clinical management.

## 2. Liquid Biopsy and HER2-Positive Breast Cancer

Using solid biopsies to identify *ERBB2* amplifications indicative of HER2 positive tumours and employing targeted HER2 therapies is nowadays well established in the clinics. Solid biopsies can provide information about tumour histology, tumour subtype as well as its molecular profile, which serve for predictive and prognostic purposes and can thus guide treatment planning with relatively optimal cost effectiveness. However, there is a lack of alternative biomarkers to predict the response or resistance to HER2-targeted therapies. Moreover, solid biopsies have several other limitations such as tissue availability/reachability and/or the impossibility to obtain enough tissue to faithfully portray the bulk of a given tumour or multiple metastatic sites. In this regard, strategies to molecularly reclassify advanced cancers aiming to identify novel treatment options are becoming popular (e.g., identification of resistance mutations to EGFR inhibitors in lung cancer [21] or *PIK3CA* aberrations in previously treated advanced BC [22]). Moreover, solid biopsies are frequently incompatible with longitudinal patient monitoring [23].

**Figure 1:**
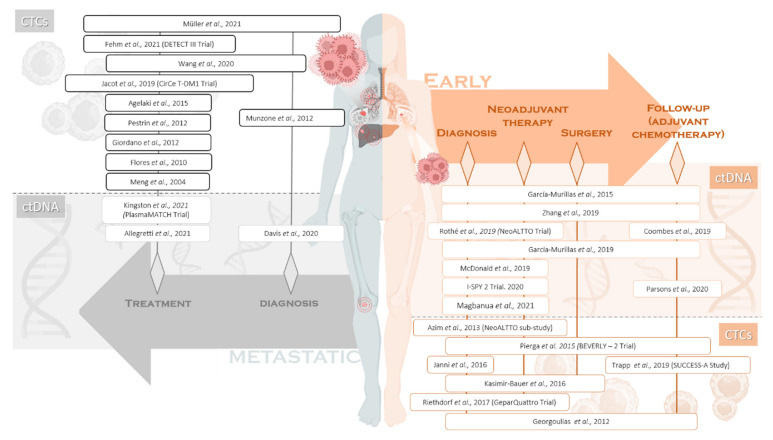
Schema depicting the reviewed studies and their focus on disease presentation and treatment stage. At clinical presentation, breast cancer (BC) patients can be divided into early (right part) and metastatic (left part) BC patients. The disease settings in patients’ clinical management are specified within the arrows. The studies included in this review are shown based on the investigated treatment stage as well as the employed liquid biopsy analyte. CTCs, circulating tumour cells; ctDNA, circulating tumour DNA.

On the other hand, liquid biopsy is a minimally invasive methodology permitting the collection and study of multiple circulating tumour components released into the bloodstream by necrosis, apoptosis or actively by tumour cells. These components, termed tumour circulome, can be divided into circulating tumour DNA (ctDNA), circulating tumour cells (CTCs), circulating cell-free RNA (cfRNA), tumour-educated platelets (TEPs), extracellular vesicles (EVs) and a wide spectrum of proteins and metabolites amongst others [24]. Liquid biopsy represents a tool that could potentially be used to obtain the complete molecular picture of a tumour, including HER2-positive BC, and perform treatment response monitoring, pathological complete response (pCR) and outcome prediction and/or screening for early tumour identification in asymptomatic individuals [25]. Herein, we review the most relevant studies employing liquid biopsy, specifically focused on ctDNA and CTCs both in early and metastatic HER2-positive BC (Figure 1).

## 3. Circulating Tumour DNA in HER2-Positive Early Breast Cancer

Circulating tumour DNA in the early BC setting has been demonstrated to represent a valid tool to predict response to treatment as well as disease relapse prior to its clinical manifestation, the so-called minimal residual disease (MRD) [24,26,27].

In this regard, García-Murillas and colleagues [26] were pioneers in tracking somatic mutations in plasma to detect early relapses using digital droplet PCR (ddPCR) (Table 1). In a subsequent extended study including all BC subtypes, these investigators observed that 29 out of 144 studied patients relapsed, and 23/29 had ctDNA detectable in blood prior to clinical recurrence. They also observed a lead time between ctDNA detection and clinical relapse of 10.7 months (Table 1) [28]. On top of that, ctDNA detection during follow-up was highly prognostic in all BC subtypes. When studying ctDNA levels in pretreatment plasma samples, they observed that HER2-positive patients showed median ctDNA amount of 0.81 copies/mL (range 0–5.4), intermediate between triple negative BC (TNBC) with the highest levels (median 4.96 copies/mL, range 0–17.0) and hormone-receptor (HR)-positive tumours with the lowest ctDNA levels (median 0 copies/mL, range 0–4.4) [28].

In a similar study, Coombes and colleagues [29] devised a personalised assay to track up to 16 somatic mutations in the plasma of early breast cancer patients as biomarkers of ctDNA and MRD. The authors detected ctDNA in 89% of the relapsed patients (16/18). Importantly, the assay showed 100% sensitivity among HER2-positive patients. In this study, all ctDNA-positive patients relapsed at latest 50 months after surgery, but ctDNA indicative of relapse was detected up to 2 years prior to clinical recurrence. When stratifying by subtype, the median lead time was the shortest in case of HER2-positive patients, followed by an intermediate value of TNBC and the longest lead time of hormone receptor-positive patients. Therefore, HER2-positive tumours seem to be the most challenging in terms of plasma ctDNA detection before relapsing clinically. However, this study is rather limited by the number of included patients, and further investigations are needed in this regard (Table 1). An additional study also uncovered the utility of personalised next-generation sequencing (NGS) panels to detect ultralow ctDNA amounts in blood. The authors achieved 91% and 53% sensitivity at mutant allele fractions of 0.003% and 0.0003%, respectively, with 96% specificity by using their novel and high-sensitive methodology called targeted digital sequencing (TARDIS). They also demonstrated high accuracy in detecting molecular response and residual disease during neoadjuvant chemotherapy (NAC). Despite these impressive results, the included HER2-positive patients were remarkably low (7 out of 33). Therefore, increasing this patient population is imperative to decipher the real impact of their novel NGS methodology in HER2-positive BC patients (Table 1) [30]. Similarly, Parsons and colleagues [31] designed an ultrasensitive blood test to detect MRD tracking hundreds of patient-specific mutations to detect ctDNA in blood draws. They demonstrated a clinical sensitivity in early-stage disease of 23% at postsurgery and 19% one year after intervention. Additionally, they showed an association between MRD detection and relapse (HR = 20.8 [95% CI: 7.3–58.9]). However, they did not observe correlation between MRD detection and BC subtype, including HER2-positive cases, because of the insufficient number of patients for each subtype (Table 1).

A translational substudy of the NeoALTTO clinical trial investigated the utility of ctDNA to evaluate treatment response and predict the outcome to anti-HER2-targeted NAC [32]. The authors measured ctDNA amounts at baseline (pre-treatment), two weeks after starting NAC and just before surgery. It is important to highlight that the reported amounts of ctDNA were probably heavily affected by the selection of genes to be studied therein (*TP53* and *PIK3CA*). ctDNA levels were correlated with clinicopathologic features, gene expression signatures, PAM50 molecular subtypes and clinical outcomes. In detail, ctDNA was detected in 41%, 20% and 5% patients before NAC, at week 2 of NAC and before surgery, respectively. The authors found a negative correlation between ctDNA levels at baseline and the probability of achieving pCR. However, they did not find any correlation between ctDNA amounts during NAC and event-free survival (EFS). Even though they did not observe correlation between ctDNA detection at any time point during treatment surveillance with posterior pCR, they pointed out that patients with a decrease in ctDNA levels during NAC were actually responding to therapy. Additionally, ctDNA positivity after one cycle of NAC correlated with shorter disease-free survival (DFS) and overall survival (OS). Importantly, despite recent evidences indicating that HER2-enriched subtype achieves increased rates of pCR after NAC when employing dual HER2 blockage [32,55], no advantage was observed in this specific study [32], probably because of the relatively small number of patients included (Table 1).

In a recent study with a similar aim, Zhang and colleagues [33] tracked tumour mutations in plasma using NGS amplification panels in a limited series of early BC cases including HER2-positive tumours before and after NAC. The authors observed that patients with ctDNA detectable prior to chemotherapy that become negative afterwards mainly bear basal-like or HER2-enriched tumours. Thus, they confirmed that these two BC intrinsic subtypes are more sensitive to chemotherapy (Table 1). With respect to pCR, it was already demonstrated that HER2-tumours are less likely to achieve pCR when treated with anti-HER2 therapies if they bear mutations in *PIK3CA*, which is a downstream member of the HER2 signalling cascade [56,57]. Interestingly, there are several clinical trials trying to investigate this issue [58] as well as to assess the utility of ctDNA in predicting therapy outcome.

A final example demonstrating ctDNA detection utility to predict the response to NAC is the phase II clinical trial I-SPY 2 [59]. This clinical study includes around 1000 patients of all subtypes that received standard NAC alone or in combination with an AKT1 inhibitor (MK-2206). In a translational research substudy, Magbanua and colleagues [34] used ctDNA detection to predict pCR and risk of metastatic recurrence in 84 high-risk early BC patients, 23% of whom had HER2-positive tumours. In total, 291 plasma samples collected at pre-, during and post-NAC were included. Overall, patients remaining ctDNA positive 3 weeks after initiation of chemotherapy were more prone to have a residual disease after NAC compared to those with undetectable ctDNA. After NAC, 100% of patients that achieved pCR were ctDNA negative. The patients without pCR but also ctDNA negative had a fairly good outcome similar to those with pCR. However, ctDNA positive patients that did not achieve pCR had an increased risk of metastatic recurrence. Interestingly, the rate of ctDNA positivity was the highest for HER2-enriched subtype, as well as for TNBC and larger tumours. Moreover, while 47.4% of HER2-positive cases achieved pCR, this subtype also encompassed the smaller proportion of cases that did not reach pCR and were ctDNA negative. Nonetheless, no subtype-specific prognostic impact of pCR was detected in the study, probably due to the low number of included patients (Table 1).

## 4. Circulating Tumour DNA in HER2-Positive Metastatic Breast Cancer

Liquid biopsy is also an excellent tool to minimal-invasively characterise advanced BC, obtaining a more complete genomic landscape of a tumour and its metastases. In this regard, the plasmaMATCH clinical trial in the UK [35] analysed an extensive cohort of metastatic BC spanning all subtypes and employed circulating-free DNA (cfDNA) sequencing to define genomic profiles. Additionally, the data from tissue sequencing were used for comparison, and the association with clinical and pathological characteristics of advanced BC was also performed to decipher causal processes generating metastatic BC diversity. In detail, the investigators observed that the number of mutations (single-nucleotide variants (SNV) and indels), as well as their variant allele frequencies (VAF) increased with lines of treatment. Indeed, HER2-positive tumours harboured more mutations in the *ERBB2* gene in those patients, who underwent more lines of treatment with anti-HER2-targeted drugs. Thus, the acquisition of HER2 mutations as consequence of anti-HER2 therapies, when identified by liquid biopsy, can be an interesting strategy for the detection of HER2-positive-resistant disease. In this regard, it had been previously demonstrated that HER2 positivity in blood, by characterizing *ERBB2* copy number status employing cfDNA sequencing, can be used to stratify responders and nonresponders to HER2-targeted therapy [60,61]. Additionally, the utility of liquid biopsy to identify *ERBB2* copy number changes was highlighted in this work. Interestingly, *ERBB2* copy number gains detectable in blood corresponded well with findings from the tumour tissue in patients with HER2 positivity. In this study, the authors set a plasma *ERBB2* copy number threshold of >2.0 copies to identify *ERBB2* amplification in a given patient with 50% sensitivity and 98% specificity. Clearly, sensitivity remains an unresolved issue demanding continual ctDNA study to determine HER2 status and to identify the minority of patients who acquire *ERBB2* amplification at relapse. Finally, the identification of subclonal mutations in *ERBB2* in HER2-positive patients should lead to their inclusion in clinical trials to investigate whether these patients could benefit from HER2 tyrosine kinase inhibitors (Table 1). In an additional study, Andrew A. Davis [36] and colleagues studied the landscape of metastatic BC using a fixed-commercial NGS panel in ctDNA. A total of 255 patients with metastatic BC were included, from which 75 patients were HER2-positive, including the HR-positive and negative cases. In this HER2-positive cohort, the most common SNVs were located in *TP53* (38), *PIK3CA* (23), and *ERBB2* (15) with *ERBB2* as the most frequently encountered copy number variations (CNV) in the cohort (33). The median mutant allele frequency (MAF) for HER2-positive tumours was 2.6 (IQR 0.3–8.0), the lowest amongst all subtypes. The authors did not observe differences in number of alterations amongst the subtypes: HR+ (median 4, IQR 2–8), HER2+ (median 4.5, IQR 2–7), and TNBC (median 5, IQR 3–7). *ERBB2* copy number alterations (CNAs) or CNV were detected in 27/28 (96.4%) of HER2-positive patients. On the other hand, the authors found mutations in *ERBB2* in 16/17 HR+ HER2-, 8/35 HER2-positive and 3/3 in TNBC cases. The authors indicated that this high proportion of *ERBB2* mutations in HR-positive patients indicates the need to assess for resistant mutations in this population and highlighted the current limitation to detect CNAs in ctDNA due to the very low tumour fraction found in plasma (Table 1). In a recent interesting study, Allegretti and colleagues investigated the genomic effect of trastuzumab emtansine (T-DM1) in HER2-positive metastatic BC patients employing liquid biopsy [37]. They performed NGS and ddPCR in an extensive set of plasma samples aiming to decipher the genetic landscape of resistant disease. Interestingly, the authors described a switch in the HER2-positive disease provoked by treatment and associated with different clinical responses. They observed a depletion of certain genetic aberrations (e.g., *PIK3CA* and *ERBB2*) and acquisition of other targetable mutations (e.g., *ESR1, MYC* and *FGFR1*) (Table 1). These results demonstrated the key role of liquid biopsy to shed light over cancer evolution during treatments as well as to detect resistance and novel targetable mutations.

## 5. Circulating Tumour Cells in HER2-Positive Early Breast Cancer

CTCs can be found in the circulation of cancer patients and serve as an important source of tumour-related information and disease monitorisation [62]. CTCs detection has been convincingly validated as a biomarker of the worse outcome in both early and metastatic BC [24]. The BEVERLY-2 study [38] is a relevant clinical trial focusing on the non-metastatic HER2-positive invasive BC, which encompassed CTC detection and pCR and provided validating evidence of their combined prognostic impact on DFS assessment. This is an open-label, single-arm, multicentre phase II study that adds neoadjuvant and adjuvant bevacizumab to the standard-of-care regimen. Of note, the prognostic value of CTCs and circulating endothelial cells in a survival analysis after 3 years of follow-up was described therein. In this study, CTC positivity was set to ≥1 CTC per 7.5 mL of blood. The detection of CTCs was not associated with patients’ clinical or pathological characteristics. Of note, 46% of the patients were CTC-positive at least once between baseline sampling to presurgery. A high percentage of patients (35%) had detectable CTCs at baseline and the positivity decreased to 7% before surgery due to NAC. CTCs detection during NAC was associated with significant reduction in DFS (CTC-positive patients had DFS of 54% compared to DFS of 83% in patients with no CTCs), but there was no association with pCR. Moreover, patients with no CTCs throughout neoadjuvant stages had a 96% OS compared to 83% for those with ≥1 CTC. When CTC detection was combined with pCR for prognostic stratification, the patients with baseline CTC counts of <1/7.5 mL (CTC negative patients), who achieved pCR, showed excellent prognosis, while those with a baseline CTC of ≥1/7.5 mL and no pCR were at higher risk of relapse. In detail, 3–4 weeks after surgery, 13.2% of included patients were CTC positive. Additionally, in an exploratory analysis, the authors observed that 3–4 weeks after surgery, CTCs were detected in 4% of patients achieving pCR and in 31% that had not. At the end of adjuvant treatments, ≥1 CTC/7.5 mL were detected in 20.7% of the patients, but the mean CTC count in the cohort was 0.3 CTC/7.5 mL. No further differences were discovered after 1 year of adjuvant therapy in the studied patients. Studying the impact of CTCs detection on DFS, the researchers observed that the 3-year DFS in patients with ≥ 1 CTC/7.5 mL at baseline was significantly lower than for patients with no CTC detected in pretreatment samples. However, they did not observe the association between CTCs and DFS in other blood time points. Additionally, patients with CTCs detected at any point during NAC presented a significant reduction in DFS (Table 1).

An additional publication describing results from the GeparQuattro study [39], which included a mixture of BC subtypes, again postulated that CTC-negative patients achieving pCR showed the best prognosis in contrast to those CTC-positive with a suboptimal response to therapy, who had higher risk of recurrence. Moreover, patients with <2 CTCs without pCR had an intermediate risk of metastatic disease. Specifically, for HER2-positive BC, the GeparQuattro trial defined that the detection of ≥2 CTCs/7.5 mL was associated with a reduced DFS in a multivariate analysis, suggesting that some of these preoperatively detected CTCs are resistant to therapies and lead to subsequent relapse (Table 1). Indeed, the presence of subclones composed of epithelial-to-mesenchymal transition (EMT)-like CTCs and stem cell-like CTCs was described to provoke resistance to conventional oncologic treatments [63,64,65], including NAC [40,66] (Table 1). Finally, the NeoALTTO phase III trial [41] also studied CTC fluctuation during the therapy of HER2-positive patients. While no significant decrease in CTC counts was achieved during NAC, a tendency towards lower pCR rates was observed in patients with detectable CTCs. This study was likely underpowered, but a meta-analysis could help to uncover interesting associations (Table 1).

In addition to the previously mentioned, CTCs detection was also employed in the adjuvant setting to correlate CTCs positivity with DFS and OS. In this context, the SUCCESS A clinical trial is a multicenter, open-label, phase III study comparing three adjuvant chemotherapy regimens and two schemes of adjuvant treatment with bisphosphonate (2 vs. 5 years of zoledronate) for early-stage high-risk BC patients [67]. CTC presence was assessed before and 2 years after chemotherapy [42]. The investigators observed notable differences in the correlation analyses between CTCs presence and DFS and OS when considering the different BC subtypes. In luminal A-like, luminal B-like and TNBC tumours, the detection of CTCs 2 years after chemotherapy was associated with decreased OS. By contrast, there was no prognostic value of CTCs detection during follow-up in patients with HER2-type tumours, contradictory to other studies (Table 1) [43,45,46]. The authors pointed to possible carryover effects, provoked by anti-HER2 targeted treatments, which can explain this phenomenon [68]. Additional information will likely be obtained through the SUCCESS B trial, an open-label, multicentre and randomised phase III study which only includes HER2-positive BC patients.

Finally, it has been demonstrated that the presence of HER2-positive CTCs in early BC patients and showed that this positivity is an independent factor from HER2-status in the primary tumour [69,70,71,72]. In an interesting study [44], it was demonstrated that patients with HER2-negative tumours but positive CTCs treated with trastuzumab showed reduced probability of relapse and improved disease-free interval. These findings could indicate that targeting HER2-positive CTCs is a suitable strategy to avoid relapses in early BC patients. In this regard, it is important to adapt isolating antibody-based platforms to specifically detect HER2-positive CTCs population. Instead, methodologies based on CTCs cell-size couple with approaches to identify HER2-positivity at protein, RNA or DNA level could represent an excellent alternative.

## 6. Circulating Tumour Cells in HER2-Positive Metastatic Breast Cancer

In the metastatic BC setting, it is crucial to decipher disease’s evolution especially related to resistance to treatments or the appearance of novel tumour clones. These processes are well documented in metastatic BC patients treated with hormone therapy, where estrogen receptor 1 (*ESR1*) mutations are responsible for treatment resistance [73]. CTCs detection can offer not only information regarding their quantity but also their genomic/transcriptomic characterisation [62,74]. The molecular interrogation of these cells can provide a molecular snapshot of the whole disease, even when metastatic site is not easily accessible by conventional biopsies [75].

In metastatic BC, it has been demonstrated that HER2 status may change between primary tumours and CTCs [20,47]. It has been also demonstrated that patients with HER2-positive tumours can present HER2-negative CTCs in their blood. Moreover, the detection of HER2-positive CTCs is not infrequent in HER2-negative tumours, as specified bellow, and could indicate worse clinical outcome. A small-series study, led by Flores and colleagues [48], reported that 33% of metastatic BC patients with HER2-negative disease had HER2-amplified CTCs. Similar CTC findings were observed in the CirCE T-DM1 trial [49] and in the plasmaMATCH study focused on ctDNA (Table 1) [35]

In an interesting study, Wang and colleagues investigated the impact of anti-HER2 therapy in patients with HER2-negative tumours but positive CTCs [50]. They observed that these patients with ≥2 HER2-positive CTCs showed less survival and better benefits from anti-HER2 therapies. Moreover, in a follow-up analysis, those patients changing between ≥2 to <2 HER2-positive CTCs presented better survival. Similarly, another study explored the effect of lapatinib in patients with HER2-positive CTCs but HER2-negative tumours [51]. The authors reported that 7 out of 96 patients with detected CTCs presented HER2-positivity. However, they did not observe tumour response to lapatinib in this patient population but only one patient with disease stabilisation. By contrast, Agelaki and colleagues [52] also treated patients with HER2-negative advanced tumours but HER2-positive CTCs with lapatinib. They observed a decrease in the HER2-positive CTCs number in patients with disease stabilisation but also in the median number of detected CTCs per patient. However, no objective responses were observed, probably because of the low number of patients included in the study. Moreover, the DETECT III study compared standard therapy alone or in combination with anti-HER2 targeted therapy (lapatinib) in patients with HER2-negative metastatic BC and HER2-positive CTCs [53]. Their results showed favourable outcomes after combined targeted treatment indicated by early decline of CTC counts. Still, this clearance was not significant compared to the standard arm [57.1% vs. 50.0%; *p* = 0.63]. Of note, patients in the lapatinib arm showed a tendency towards a better progression-free survival (PFS) and a significantly improved OS by univariate (HR 0.54; 95% CI, 0.34–0.86; *p* = 0.008) and multivariate (HR 0.55; 95% CI, 0.34–0.90; *p* = 0.016) cox regression analysis when compared to those in the standard arm. The CTC clearance at the end of treatment was not associated with OS, although better OS was observed in patients with no evidence of CTCs at first follow-up compared to patients with detectable CTCs (HR 0.36; 95% CI, 0.17–0.76; *p* = 0.005) (Table 1). Finally, in a very recent research article also within the DETECT program, the authors analysed the HER2 status of CTCs in patients HER2-negative tumours and its clinical significance [54]. Herein, CTCs HER2 status was analysed in 1159 CTCs-positive patients. They observed that patients with estrogen and progesterone-positive status were more prone to present strong-stained HER2 CTCs. The authors also demonstrated the association of ≥1 CTCs strong-stained for HER2 with shorter OS but not between the proportion of HER2-positive CTCs and clinical outcome. The association with OS is lost when including moderate-stained HER2 CTCs. Importantly, CTCs HER2 status was not significantly associated to PFS in this cohort.

## 7. Conclusions and Future Perspectives in HER2-Positive Breast Cancer and Liquid Biopsy

In this review, we showed several studies focused on ctDNA and CTCs demonstrating potential for a range of different clinical applications in BC (Table 1 and Figure 1) but also specifically in HER2-positive tumours. Despite other circulating tumour components such as exosomes or micro-RNAs (miRNAs) are being investigated [24], there are no publications available demonstrating their importance in HER2-positive BC.

While most of the studies reviewed thus far are based on limited sample sets, it is already clear that liquid biopsy and ctDNA detection in the HER2-positive early BC setting can provide prognostic information regarding both the response to NAC and early relapse. In this regard, ctDNA monitoring could be a tool to modulate NAC schedules and avoid overtreatment in low-risk patients or increase dosage in those with suboptimal responses. In the adjuvant setting, the detection of MRD and recurrence before it is detectable clinically could crucially improve BC patients’ survival by the timely starting of adjuvant therapy as well as by preventing overtreatment in patients with a lower risk of relapse. In this regard, large clinical studies employing ctDNA detection for clinical decision-making and personalisation are needed for the final validation of this methodology. Ideally, these studies employ ultrasensitive methodologies to detect ultralow levels of ctDNA.

Likewise, more studies are needed to determine the role of ctDNA detection in the metastatic HER2-positive BC. The development of novel methodologies to sensitively track *ERBB2* amplifications and mutations in blood and characterise disease evolution, resistance and the acquisition of HER2-positivity in tumours previously HER2-negative is paramount to improve the clinical manage of BC patients.

More research also warrants to clarify the possible application of CTCs counts in clinics both in the early and metastatic setting. During NAC, certain associations between CTCs detection and worse outcomes were already observed. However, important discrepancies can be found between CTCs counts and response to NAC. In the same manner, no conclusive results exist to clarify whether CTCs detection in early HER2-positive BC during adjuvant therapy would be beneficial for patients’ stratification. In addition, a shift between HER2-negative to positive disease at advanced disease stages was demonstrated using CTCs detectable in the blood of some patients with HER2-negative tumours. Moreover, changes in CTCs counts and clinical benefits were addressed in these patients when they are treated with anti-HER2 therapies. However, there is a need to develop studies including a higher number of patients to achieve consistent conclusions. It is necessary to develop novel and highly sensitive technologies to detect as well as to characterise HER2-negative or positive CTCs in the metastatic BC setting. Overall, it is important to shed light over the variability aspect between techniques as a factor behind the contradictory observations in the different studies. On top of that, it is crucial to understand how the disease is evolving and detect early resistance to be able to efficiently treat it. The advanced disease can provide us with high CTCs numbers that, unlike ctDNA, potentially offer deep insights about the disease transcriptomics and genomics.

## Figures and Tables

**Table 1:** Reviewed liquid biopsy studies in early and metastatic breast cancer.

Study	Analyte	Methodology	HER2-Positive (*n*)	Findings	References
ctDNA in HER2-positive early BC					
García-Murillas et al., 2015	ctDNA	ddPCR	21	- Correlation between risk of relapse and ctDNA detection. - ctDNA sequencing could serve to characterise the genetic landscape of MRD.	[26]
García-Murillas et al., 2019		ddPCR	41	- Improved relapse lead time over clinical identification of 10.7 months by ctDNA detection. - HER2-positive tumours show intermediate ctDNA abundance (0.81 copies/mL).	[28]
Coombes et al., 2019		Patient-specific panel and ultradeep sequencing	8	- ctDNA detection rate of 89%. - ctDNA detection in HER2-positive patients show 100% sensitivity.	[29]
McDonald et al., 2019		TARDIS	7	- Ten years relapse-free survival is 95% for HER2-positive patients.	[30]
Parsons et al., 2020		NGS with UMIs	25	- No correlation observed between MRD detection and tumour subtype, including HER2-positive.	[31]
Rothé et al., 2019		ddPCR	455	- Highest pCR rates for patients with HER2-enriched subtypes and undetectable ctDNA.	[32]
Zhang et al., 2019		Large NGS panels	27	- ctDNA detection rate of 74.2% in early BC. - Patients with ctDNA detected before chemotherapy that becomes negative after that are mainly basal-like or HER2-enriched tumours. - Basal-like and HER2-enriched tumour subtypes are more sensitive to chemotherapy.	[33]
Magbanua et al., 2021		Patient-specific panel and ultradeep sequencing	19	- ctDNA positivity is higher in HER2-enriched tumours, TNBC and larger tumours. - 47.4% of HER2-positive cases achieved pCR.	[34]
ctDNA in HER2-positive metastatic BC					
Kingston et al., 2021		Guardant360 cfDNA assay-ddPCR	72	- More mutations are found in the *ERBB2* gene in patients with more anti-HER2 targeted lines of treatment. - Exclusive *ERBB2* mutations in HER2-positive tumours.	[35]
Davis et al., 2020		Guardant360	75	- *TP53*, *PIK3CA* and *ERBB2* were the most common mutated genes in HER2-positive tumours. - *ERBB2* was the most frequent CNV in the HER2-positive cohort. - CNVs in *ERBB2* were detected in 96.4% of HER2-positive patients.	[36]
Allegretti et al., 2021		ddPCR and NGS	20	- Molecular subtype switch as consequence of treatment. - Detection of new targetable alterations (*ESR1, MYC* and *FGFR1*).	[37]
CTCs in HER2-positive early BC					
Pierga et al., 2015	CTCs	CellSearch^®^	52	- Detectable CTCs are associated with shorter 3-years DFS and OS. - In the neoadjuvant setting: ⚬ Combining CTC detection and pCR those patients with baseline CTCs counts of <1/7.5 mL and pCR show excellent prognosis yet those with baseline CTC of ≥1/7.5 mL and no pCR are at higher risk of relapse. ⚬ Patients presenting ≥1 CTC have a DFS of 54% and OS of 96% compared with a DFS and OS of 83% in patients without CTCs detected.	[38]
Riethdorf et al., 2017		CellSearch^®^	59/63	- ≥1 CTC and ≥2 CTCs before NAC is associated with reduced DFS and OS. - CTCs-negative patients with pCR show the best prognosis. - CTC-positive patients with decreased tumour response correlate with high risk of relapse. - For HER2-positive BC, the detection of ≥2 CTCs/7.5 mL show reduced DFS.	[39]
Kasimir-Bauer et al., 2016		AdnaTest ^®^	56	- CTCs are detected in 24% and 8% of BC patients before and after NAC, respectively. - There was no association between CTCs detection and PFS or OS. - Therapy-resistant CTCs detected after NAC could indicate worse outcome.	[40]
Azim et al., 2013		CellSearch^®^	51	- No significant CTC counts decrease during NAC is associated with lower pCR rates	[41]
Trapp et al., 2019		CellSearch^®^	279	- CTCs detection have prognostic features in patients with luminal A-like, luminal B-like or TNBC tumours but not in the HER2-positive subtype.	[42]
Janni et al., 2016		CellSearch^®^	688	- A decreased OS is associated with CTCs detection two years after adjuvant chemotherapy in Luminal A-like, luminal B-like and TNBC tumours. - CTC status has prognostic relevance in HR-negative/HER2-positive tumours but not in HR-positive/HER2-positive. - A high prognostic value when HER2-positive patients are combined independently of the HR status. - No demonstration of a prognostic value for CTCs detection during follow-up in patients with HER2-type tumours.	[43]
Georgoulias, et al., 2012		Cytocentrifugation + IF	-	- CK+/HER2+ CTCs detected in 90% of the patients. - Anti-HER2 therapy decrease the number of CTCs, the risk of relapse and increase DFS	[44]
CTCs in HER2-positive metastatic BC					
Munzone et al., 2012	CTCs	CellSearch^®^	53	- No demonstrated prognostic value for CTCs detection during follow-up in patients with HER2-type tumours.	[45]
Giordano, et al., 2012		CellSearch^®^	101	- CTCs detection is strongly associated with OS prediction in all MBC subtypes excluding HER2-positive patients treated with anti-HER2 therapy.	[46]
Meng, et al., 2004		Ferrofluids + FISH	-	- HER2 expression in ten CTCs was enough to diagnose the *ERBB2* gene status. - 37.5% of cases with HER2-negative primary tumours have *ERBB2* amplified in their CTCs during progression.	[47]
Flores et al., 2010		CellSearch^®^/CellSearch Profile Kit (PFK) + FISH	45	- 33% of metastatic BC patients with HER2-negative disease presented HER2-amplified CTCs	[48]
Jacot et al., 2019		CellSearch^®^	-	- 9.1% of HER2-negative MBC patients with ≥1 CTC/7.5 mL presented ≥1 HER2-amplified CTC.	[49]
Wang et al., 2020		CellSearch^®^	-	- High-risk HER2+ CTC-patients had shorter survival and higher progression risk. - Anti-HER2 therapy increased PFS. - PFS was higher in patients switching from high- to low-risk HER2+ CTCs during treatment.	[50]
Pestrin et al., 2012		CellSearch^®^ + IF	-	- No benefit was observed when patients with HER2-negative tumours and HER2-positive CTCs were treated with anti-HER2 therapy.	[51]
Agelaki et al., 2015		IF	2	- Lapatinib substantially decreases the number of HER2-positive CTCs in patients with HER2-negative tumours only in those with disease stabilisation.	[52]
Fehm et al., 2021		CellSearch^®^+IHC or FISH	-	- Patients with HER2-negative tumours presented HER2-positive CTCs. - CTCs detection is not associated with OS. - Patients with no CTCs detected at first follow-up had better OS. - Lapatinib treatment is effective to decrease HER2-positive CTC independently of HER2 status of the primary tumour.	[53]
Müller et al., 2021		CellSearch^®^	-	- The presence of ≥1 strong-stained HER2 CTCs is associated with shorter OS but not with better PFS. - The association with shorter OS was not observed in patients with moderate-stained HER2 CTCs.	[54]

Liquid biopsy studies in early and metastatic breast cancer (BC) including or focusing on HER2-positive BC patients (*n*). Studies employing both circulating-tumour DNA (ctDNA) or circulating tumour cells (CTCs) were examined. HER2, human epidermal growth factor receptor 2; CTCs, circulating-tumour cells; ctDNA, circulating-tumour DNA; ddPCR, droplet-digital PCR; MRD, minimal residual disease; pCR, pathological complete response; TNBC, triple-negative breast cancer; IF, immunofluorescence; BC, breast cancer; MBC, metastatic breast cancer; NGS, next-generation sequencing; UMIS, unique molecular identifiers; cfDNA, circulating-free DNA; SNV, single nucleotide variant; CNV, copy number variant; DFS, disease-free survival; OS, overall survival; NAC, neoadjuvant chemotherapy; PFS, progression-free survival; IHC, immunohistochemistry; and FISH, fluorescence in situ hybridisation.

## Abbreviations

Circulating-free DNA (cfDNA), circulating cell-free RNA (cfRNA), circulating tumour DNA (ctDNA), circulating tumour cells (CTCs), copy number variations (CNV), digital PCR (ddPCR), disease-free survival (DFS), epithelial-to-mesenchymal transition (EMT), event-free survival (EFS), extracellular vesicles (EVs), fluorescence in-situ hybridisation (FISH), hazard ratio (HR), hormone-receptor (HR), immunofluorescence (IF), immunohistochemistry (IHC), in situ hybridisation (ISH), micro RNAs (miRNAs), minimal residual disease (MRD), mutant allele frequency (MAF), neoadjuvant chemotherapy (NAC), next-generation sequencing (NGS), overall survival (OS), pathological complete response (pCR), progression-free survival (PFS), single-nucleotide variants (SNV), triple negative BC (TNBC), targeted digital sequencing (TARDIS), tumour-educated platelets (TEPs), unique molecular identifiers (UMIs), and variant allele frequencies (VAF).

## References

[1] Cejalvo J.M., Pascual T., Fernández-Martínez A., Brasó-Maristany F., Gomis R.R., Perou C.M., Muñoz M., Prat A. (2018). Clinical Implications of the Non-Luminal Intrinsic Subtypes in Hormone Receptor-Positive Breast Cancer. Cancer Treat. Rev..

[2] Loibl S., Poortmans P., Morrow M., Denkert C., Curigliano G. (2021). Breast Cancer. Lancet.

[3] Harbeck N., Penault-Llorca F., Cortes J., Gnant M., Houssami N., Poortmans P., Ruddy K., Tsang J., Cardoso F. (2019). Breast Cancer. Nat. Rev. Dis. Primers.

[4] Survival Rates for Breast Cancer. https://www.cancer.org/cancer/breast-cancer/understanding-a-breast-cancer-diagnosis/breast-cancer-survival-rates.html.

[5] Lüönd F., Tiede S., Christofori G. (2021). Breast Cancer as an Example of Tumour Heterogeneity and Tumour Cell Plasticity during Malignant Progression. Br. J. Cancer.

[6] Perou C.M., Sørlie T., Eisen M.B., van de Rijn M., Jeffrey S.S., Rees C.A., Pollack J.R., Ross D.T., Johnsen H., Akslen L.A. (2000). Molecular Portraits of Human Breast Tumours. Nature.

[7] Prat A., Perou C.M. (2011). Deconstructing the Molecular Portraits of Breast Cancer. Mol. Oncol..

[8] Cheang M.C.U., Chia S.K., Voduc D., Gao D., Leung S., Snider J., Watson M., Davies S., Bernard P.S., Parker J.S. (2009). Ki67 Index, HER2 Status, and Prognosis of Patients with Luminal B Breast Cancer. J. Natl. Cancer Inst..

[9] Harbeck N. (2015). Insights into Biology of Luminal HER2 vs. Enriched HER2 Subtypes: Therapeutic Implications. Breast.

[10] Arriola E., Marchio C., Tan D.S., Drury S.C., Lambros M.B., Natrajan R., Rodriguez-Pinilla S.M., Mackay A., Tamber N., Fenwick K. (2008). Genomic Analysis of the HER2/TOP2A Amplicon in Breast Cancer and Breast Cancer Cell Lines. Lab. Inv..

[11] Godoy-Ortiz A., Sanchez-Muñoz A., Chica Parrado M.R., Álvarez M., Ribelles N., Rueda Dominguez A., Alba E. (2019). Deciphering HER2 Breast Cancer Disease: Biological and Clinical Implications. Front. Oncol..

[12] Prat A., Chaudhury A., Solovieff N., Paré L., Martinez D., Chic N., Martínez-Sáez O., Brasó-Maristany F., Lteif A., Taran T. (2021). Correlative Biomarker Analysis of Intrinsic Subtypes and Efficacy Across the MONALEESA Phase III Studies. JCO.

[13] Wolff A.C., Hammond M.E.H., Allison K.H., Harvey B.E., Mangu P.B., Bartlett J.M.S., Bilous M., Ellis I.O., Fitzgibbons P., Hanna W. (2018). Human Epidermal Growth Factor Receptor 2 Testing in Breast Cancer: American Society of Clinical Oncology/College of American Pathologists Clinical Practice Guideline Focused Update. J. Clin. Oncol..

[14] Ferrari A., Sertier A.-S., Vincent-Salomon A., Pivot X., Pauporté I., Saintigny P., Birnbaum D., Viari A. (2016). A Phenotypic and Mechanistic Perspective on Heterogeneity of HER2-Positive Breast Cancers. Mol. Cell Oncol..

[15] Bertucci F., Ng C.K.Y., Patsouris A., Droin N., Piscuoglio S., Carbuccia N., Soria J.C., Dien A.T., Adnani Y., Kamal M. (2019). Genomic Characterization of Metastatic Breast Cancers. Nature.

[16] Nayar U., Cohen O., Kapstad C., Cuoco M.S., Waks A.G., Wander S.A., Painter C., Freeman S., Persky N.S., Marini L. (2019). Acquired HER2 Mutations in ER+ Metastatic Breast Cancer Confer Resistance to Estrogen Receptor-Directed Therapies. Nat. Genet..

[17] Amir E., Miller N., Geddie W., Freedman O., Kassam F., Simmons C., Oldfield M., Dranitsaris G., Tomlinson G., Laupacis A. (2012). Prospective Study Evaluating the Impact of Tissue Confirmation of Metastatic Disease in Patients with Breast Cancer. J. Clin. Oncol..

[18] Amir E., Clemons M., Purdie C.A., Miller N., Quinlan P., Geddie W., Coleman R.E., Freedman O.C., Jordan L.B., Thompson A.M. (2012). Tissue Confirmation of Disease Recurrence in Breast Cancer Patients: Pooled Analysis of Multi-Centre, Multi-Disciplinary Prospective Studies. Cancer Treat. Rev..

[19] Niikura N., Liu J., Hayashi N., Mittendorf E.A., Gong Y., Palla S.L., Tokuda Y., Gonzalez-Angulo A.M., Hortobagyi G.N., Ueno N.T. (2012). Loss of Human Epidermal Growth Factor Receptor 2 (HER2) Expression in Metastatic Sites of HER2-Overexpressing Primary Breast Tumors. J. Clin. Oncol..

[20] Aktas B., Kasimir-Bauer S., Müller V., Janni W., Fehm T., Wallwiener D., Pantel K., Tewes M. (2016). Comparison of the HER2, Estrogen and Progesterone Receptor Expression Profile of Primary Tumor, Metastases and Circulating Tumor Cells in Metastatic Breast Cancer Patients. BMC Cancer.

[21] Tumbrink H.L., Heimsoeth A., Sos M.L. (2021). The next Tier of EGFR Resistance Mutations in Lung Cancer. Oncogene.

[22] André F., Ciruelos E.M., Juric D., Loibl S., Campone M., Mayer I.A., Rubovszky G., Yamashita T., Kaufman B., Lu Y.-S. (2021). Alpelisib plus Fulvestrant for PIK3CA-Mutated, Hormone Receptor-Positive, Human Epidermal Growth Factor Receptor-2–Negative Advanced Breast Cancer: Final Overall Survival Results from SOLAR-1. Ann. Oncol..

[23] Kilgour E., Rothwell D.G., Brady G., Dive C. (2020). Liquid Biopsy-Based Biomarkers of Treatment Response and Resistance. Cancer Cell.

[24] Alba-Bernal A., Lavado-Valenzuela R., Domínguez-Recio M.E., Jiménez-Rodriguez B., Queipo-Ortuño M.I., Alba E., Comino-Méndez I. (2020). Challenges and Achievements of Liquid Biopsy Technologies Employed in Early Breast Cancer. EBioMedicine.

[25] Alix-Panabières C., Schwarzenbach H., Pantel K. (2012). Circulating Tumor Cells and Circulating Tumor DNA. Annu. Rev. Med..

[26] Garcia-Murillas I., Schiavon G., Weigelt B., Ng C., Hrebien S., Cutts R.J., Cheang M., Osin P., Nerurkar A., Kozarewa I. (2015). Mutation Tracking in Circulating Tumor DNA Predicts Relapse in Early Breast Cancer. Sci. Transl. Med..

[27] Li S., Lai H., Liu J., Liu Y., Jin L., Li Y., Liu F., Gong Y., Guan Y., Yi X. (2020). Circulating Tumor DNA Predicts the Response and Prognosis in Patients with Early Breast Cancer Receiving Neoadjuvant Chemotherapy. JCO Precis. Oncol..

[28] Garcia-Murillas I., Chopra N., Comino-Méndez I., Beaney M., Tovey H., Cutts R.J., Swift C., Kriplani D., Afentakis M., Hrebien S. (2019). Assessment of Molecular Relapse Detection in Early-Stage Breast Cancer. JAMA Oncol..

[29] Coombes R.C., Page K., Salari R., Hastings R.K., Armstrong A., Ahmed S., Ali S., Cleator S., Kenny L., Stebbing J. (2019). Personalized Detection of Circulating Tumor DNA Antedates Breast Cancer Metastatic Recurrence. Clin. Cancer Res..

[30] McDonald B.R., Contente-Cuomo T., Sammut S.-J., Odenheimer-Bergman A., Ernst B., Perdigones N., Chin S.-F., Farooq M., Mejia R., Cronin P.A. (2019). Personalized Circulating Tumor DNA Analysis to Detect Residual Disease after Neoadjuvant Therapy in Breast Cancer. Sci. Transl. Med..

[31] Parsons H.A., Rhoades J., Reed S.C., Gydush G., Ram P., Exman P., Xiong K., Lo C.C., Li T., Fleharty M. (2020). Sensitive Detection of Minimal Residual Disease in Patients Treated for Early-Stage Breast Cancer. Clin. Cancer Res..

[32] Rothé F., Silva M.J., Venet D., Campbell C., Bradburry I., Rouas G., de Azambuja E., Maetens M., Fumagalli D., Rodrik-Outmezguine V. (2019). Circulating Tumor DNA in HER2-Amplified Breast Cancer: A Translational Research Substudy of the NeoALTTO Phase III Trial. Clin. Cancer Res..

[33] Zhang X., Zhao W., Wei W., You Z., Ou X., Sun M., Yin Y., Tang X., Zhao Z., Hu C. (2019). Parallel Analyses of Somatic Mutations in Plasma Circulating Tumor DNA (CtDNA) and Matched Tumor Tissues in Early-Stage Breast Cancer. Clin. Cancer Res..

[34] Magbanua M.J.M., Swigart L.B., Wu H.-T., Hirst G.L., Yau C., Wolf D.M., Tin A., Salari R., Shchegrova S., Pawar H. (2021). Circulating Tumor DNA in Neoadjuvant-Treated Breast Cancer Reflects Response and Survival. Ann. Oncol..

[35] Kingston B., Cutts R.J., Bye H., Beaney M., Walsh-Crestani G., Hrebien S., Swift C., Kilburn L.S., Kernaghan S., Moretti L. (2021). Genomic Profile of Advanced Breast Cancer in Circulating Tumour DNA. Nat. Commun..

[36] Davis A.A., Jacob S., Gerratana L., Shah A.N., Wehbe F., Katam N., Zhang Q., Flaum L., Siziopikou K.P., Platanias L.C. (2020). Landscape of Circulating Tumour DNA in Metastatic Breast Cancer. EBioMedicine.

[37] Allegretti M., Fabi A., Giordani E., Ercolani C., Romania P., Nisticò C., Gasparro S., Barberi V., Ciolina M., Pescarmona E. (2021). Liquid Biopsy Identifies Actionable Dynamic Predictors of Resistance to Trastuzumab Emtansine (T-DM1) in Advanced HER2-Positive Breast Cancer. Mol. Cancer.

[38] Pierga J.-Y., Petit T., Lévy C., Ferrero J.-M., Campone M., Gligorov J., Lerebours F., Roché H., Bachelot T., Charafe-Jauffret E. (2015). Pathological Response and Circulating Tumor Cell Count Identifies Treated HER2+ Inflammatory Breast Cancer Patients with Excellent Prognosis: BEVERLY-2 Survival Data. Clin. Cancer Res..

[39] Riethdorf S., Müller V., Loibl S., Nekljudova V., Weber K., Huober J., Fehm T., Schrader I., Hilfrich J., Holms F. (2017). Prognostic Impact of Circulating Tumor Cells for Breast Cancer Patients Treated in the Neoadjuvant “Geparquattro” Trial. Clin Cancer Res..

[40] Kasimir-Bauer S., Bittner A.-K., König L., Reiter K., Keller T., Kimmig R., Hoffmann O. (2016). Does Primary Neoadjuvant Systemic Therapy Eradicate Minimal Residual Disease? Analysis of Disseminated and Circulating Tumor Cells before and after Therapy. Breast Cancer Res..

[41] Azim H.A., Rothé F., Aura C.M., Bavington M., Maetens M., Rouas G., Gebhart G., Gamez C., Eidtmann H., Baselga J. (2013). Circulating Tumor Cells and Response to Neoadjuvant Paclitaxel and HER2-Targeted Therapy: A Sub-Study from the NeoALTTO Phase III Trial. Breast.

[42] Trapp E., Janni W., Schindlbeck C., Jückstock J., Andergassen U., de Gregorio A., Alunni-Fabbroni M., Tzschaschel M., Polasik A., Koch J.G. (2019). Presence of Circulating Tumor Cells in High-Risk Early Breast Cancer During Follow-Up and Prognosis. J. Natl. Cancer Inst..

[43] Janni W.J., Rack B., Terstappen L.W.M.M., Pierga J.-Y., Taran F.-A., Fehm T., Hall C., de Groot M.R., Bidard F.-C., Friedl T.W.P. (2016). Pooled Analysis of the Prognostic Relevance of Circulating Tumor Cells in Primary Breast Cancer. Clin. Cancer Res..

[44] Georgoulias V., Bozionelou V., Agelaki S., Perraki M., Apostolaki S., Kallergi G., Kalbakis K., Xyrafas A., Mavroudis D. (2012). Trastuzumab Decreases the Incidence of Clinical Relapses in Patients with Early Breast Cancer Presenting Chemotherapy-Resistant CK-19mRNA-Positive Circulating Tumor Cells: Results of a Randomized Phase II Study. Ann. Oncol..

[45] Munzone E., Botteri E., Sandri M.T., Esposito A., Adamoli L., Zorzino L., Sciandivasci A., Cassatella M.C., Rotmensz N., Aurilio G. (2012). Prognostic Value of Circulating Tumor Cells According to Immunohistochemically Defined Molecular Subtypes in Advanced Breast Cancer. Clin. Breast Cancer.

[46] Giordano A., Giuliano M., Laurentiis M.D., Arpino G., Jackson S., Handy B.C., Ueno N.T., Andreopoulou E., Alvarez R.H., Valero V. (2012). Circulating Tumor Cells in Immunohistochemical Subtypes of Metastatic Breast Cancer: Lack of Prediction in HER2-Positive Disease Treated with Targeted Therapy. Ann. Oncol..

[47] Meng S., Tripathy D., Shete S., Ashfaq R., Haley B., Perkins S., Beitsch P., Khan A., Euhus D., Osborne C. (2004). HER-2 Gene Amplification Can Be Acquired as Breast Cancer Progresses. Proc. Natl. Acad. Sci. USA.

[48] Flores L.M., Kindelberger D.W., Ligon A.H., Capelletti M., Fiorentino M., Loda M., Cibas E.S., Jänne P.A., Krop I.E. (2010). Improving the Yield of Circulating Tumour Cells Facilitates Molecular Characterisation and Recognition of Discordant HER2 Amplification in Breast Cancer. Br. J. Cancer.

[49] Jacot W., Cottu P., Berger F., Dubot C., Venat-Bouvet L., Lortholary A., Bourgeois H., Bollet M., Servent V., Luporsi E. (2019). Actionability of HER2-Amplified Circulating Tumor Cells in HER2-Negative Metastatic Breast Cancer: The CirCe T-DM1 Trial. Breast Cancer Res..

[50] Wang C., Mu Z., Ye Z., Zhang Z., Abu-Khalaf M.M., Silver D.P., Palazzo J.P., Jagannathan G., Fellin F.M., Bhattacharya S. (2020). Prognostic Value of HER2 Status on Circulating Tumor Cells in Advanced-Stage Breast Cancer Patients with HER2-Negative Tumors. Breast Cancer Res. Treat..

[51] Pestrin M., Bessi S., Puglisi F., Minisini A.M., Masci G., Battelli N., Ravaioli A., Gianni L., Di Marsico R., Tondini C. (2012). Final Results of a Multicenter Phase II Clinical Trial Evaluating the Activity of Single-Agent Lapatinib in Patients with HER2-Negative Metastatic Breast Cancer and HER2-Positive Circulating Tumor Cells. A Proof-of-Concept Study. Breast Cancer Res. Treat..

[52] Agelaki S., Kalykaki A., Markomanolaki H., Papadaki M.A., Kallergi G., Hatzidaki D., Kalbakis K., Mavroudis D., Georgoulias V. (2015). Efficacy of Lapatinib in Therapy-Resistant HER2-Positive Circulating Tumor Cells in Metastatic Breast Cancer. PLoS ONE.

[53] Fehm T., Mueller V., Banys-Paluchowski M., Fasching P.A., Friedl T.W., Hartkopf A., Huober J., Loehberg C., Rack B., Riethdorf S. (2021). Abstract PD3-12: Efficacy of the Tyrosine Kinase Inhibitor Lapatinib in the Treatment of Patients with HER2-Negative Metastatic Breast Cancer and HER2-Positive Circulating Tumor Cells—Results from the Randomized Phase III DETECT III Trial. Cancer Res..

[54] Müller V., Banys-Paluchowski M., Friedl T.W.P., Fasching P.A., Schneeweiss A., Hartkopf A., Wallwiener D., Rack B., Meier-Stiegen F., Huober J. (2021). Prognostic Relevance of the HER2 Status of Circulating Tumor Cells in Metastatic Breast Cancer Patients Screened for Participation in the DETECT Study Program. ESMO Open.

[55] Fumagalli D., Venet D., Ignatiadis M., Azim H.A., Maetens M., Rothé F., Salgado R., Bradbury I., Pusztai L., Harbeck N. (2017). RNA Sequencing to Predict Response to Neoadjuvant Anti-HER2 Therapy: A Secondary Analysis of the NeoALTTO Randomized Clinical Trial. JAMA Oncol..

[56] Shi W., Jiang T., Nuciforo P., Hatzis C., Holmes E., Harbeck N., Sotiriou C., Peña L., Loi S., Rosa D.D. (2017). Pathway Level Alterations Rather than Mutations in Single Genes Predict Response to HER2-Targeted Therapies in the Neo-ALTTO Trial. Ann. Oncol..

[57] Majewski I.J., Nuciforo P., Mittempergher L., Bosma A.J., Eidtmann H., Holmes E., Sotiriou C., Fumagalli D., Jimenez J., Aura C. (2015). PIK3CA Mutations Are Associated with Decreased Benefit to Neoadjuvant Human Epidermal Growth Factor Receptor 2-Targeted Therapies in Breast Cancer. J. Clin. Oncol..

[58] Novartis Pharmaceuticals (2019). NeoPHOEBE: Pi3k Inhibition in Her2 OverExpressing Breast CancEr: A Phase II, Randomized, Parallel Cohort, Two Stage, Double-Blind, Placebo-Controlled Study of Neoadjuvant Trastuzumab Versus Trastuzumab + BKM120 in Combination with Weekly Paclitaxel in HER2-Positive, PIK3CA Wild-Type and PIK3CA Mutant Primary Breast Cancer.

[59] (2020). I-SPY2 Trial Consortium Association of Event-Free and Distant Recurrence–Free Survival with Individual-Level Pathologic Complete Response in Neoadjuvant Treatment of Stages 2 and 3 Breast Cancer: Three-Year Follow-up Analysis for the I-SPY2 Adaptively Randomized Clinical Trial. JAMA Oncol..

[60] Siravegna G., Sartore-Bianchi A., Nagy R.J., Raghav K., Odegaard J.I., Lanman R.B., Trusolino L., Marsoni S., Siena S., Bardelli A. (2019). Plasma HER2 (ERBB2) Copy Number Predicts Response to HER2-Targeted Therapy in Metastatic Colorectal Cancer. Clin. Cancer Res..

[61] Janjigian Y.Y., Maron S.B., Chatila W.K., Millang B., Chavan S.S., Alterman C., Chou J.F., Segal M.F., Simmons M.Z., Momtaz P. (2020). First-Line Pembrolizumab and Trastuzumab in HER2-Positive Oesophageal, Gastric, or Gastro-Oesophageal Junction Cancer: An Open-Label, Single-Arm, Phase 2 Trial. Lancet Oncol..

[62] Heidrich I., Ačkar L., Mossahebi Mohammadi P., Pantel K. (2021). Liquid Biopsies: Potential and Challenges. Int. J. Cancer.

[63] Okabe T., Togo S., Fujimoto Y., Watanabe J., Sumiyoshi I., Orimo A., Takahashi K. (2020). Mesenchymal Characteristics and Predictive Biomarkers on Circulating Tumor Cells for Therapeutic Strategy. Cancers.

[64] Bittner A.-K., Keup C., Hoffmann O., Hauch S., Kimmig R., Kasimir-Bauer S. (2020). Molecular Characterization of Circulating Tumour Cells Identifies Predictive Markers for Outcome in Primary, Triple-Negative Breast Cancer Patients. J. Cell Mol. Med..

[65] Kasimir-Bauer S., Hoffmann O., Wallwiener D., Kimmig R., Fehm T. (2012). Expression of Stem Cell and Epithelial-Mesenchymal Transition Markers in Primary Breast Cancer Patients with Circulating Tumor Cells. Breast Cancer Res..

[66] Mego M., Mani S.A., Lee B.-N., Li C., Evans K.W., Cohen E.N., Gao H., Jackson S.A., Giordano A., Hortobagyi G.N. (2012). Expression of Epithelial-Mesenchymal Transition-Inducing Transcription Factors in Primary Breast Cancer: The Effect of Neoadjuvant Therapy. Int. J. Cancer.

[67] Janni W., Friedl T.W.P., Fehm T., Müller V., Lichtenegger W., Blohmer J., Lorenz R., Forstbauer H., Bauer E., Fink V. (2018). Abstract GS1-06: Extended Adjuvant Bisphosphonate Treatment over Five Years in Early Breast Cancer Does Not Improve Disease-Free and Overall Survival Compared to Two Years of Treatment: Phase III Data from the SUCCESS A Study. Cancer Res..

[68] Ignatiadis M., Rack B., Rothé F., Riethdorf S., Decraene C., Bonnefoi H., Dittrich C., Messina C., Beauvois M., Trapp E. (2016). Liquid Biopsy-Based Clinical Research in Early Breast Cancer: The EORTC 90091-10093 Treat CTC Trial. Eur. J. Cancer.

[69] Fehm T., Becker S., Duerr-Stoerzer S., Sotlar K., Mueller V., Wallwiener D., Lane N., Solomayer E., Uhr J. (2007). Determination of HER2 Status Using Both Serum HER2 Levels and Circulating Tumor Cells in Patients with Recurrent Breast Cancer Whose Primary Tumor Was HER2 Negative or of Unknown HER2 Status. Breast Cancer Res..

[70] Brandt B., Roetger A., Heidl S., Jackisch C., Lelle R.J., Assmann G., Zänker K.S. (1998). Isolation of Blood-Borne Epithelium-Derived c-ErbB-2 Oncoprotein-Positive Clustered Cells from the Peripheral Blood of Breast Cancer Patients. Int. J. Cancer.

[71] Xu Y., Yao L., Li H., Ouyang T., Li J., Wang T., Fan Z., Lin B., Lu Y., Larsson O. (2006). Presence of ErbB2 MRNA in the Plasma of Breast Cancer Patients Is Associated with Circulating Tumor Cells and Negative Estrogen and Progesterone Receptor Status. Breast Cancer Res. Treat..

[72] Solomayer E.F., Becker S., Pergola-Becker G., Bachmann R., Krämer B., Vogel U., Neubauer H., Wallwiener D., Huober J., Fehm T.N. (2006). Comparison of HER2 Status between Primary Tumor and Disseminated Tumor Cells in Primary Breast Cancer Patients. Breast Cancer Res. Treat..

[73] Chan J.C.H., Chow J.C.H., Ho C.H.M., Tsui T.Y.M., Cho W.C. (2021). Clinical Application of Circulating Tumor DNA in Breast Cancer. J. Cancer Res. Clin. Oncol..

[74] Keller L., Belloum Y., Wikman H., Pantel K. (2021). Clinical Relevance of Blood-Based CtDNA Analysis: Mutation Detection and Beyond. Br. J. Cancer.

[75] Appierto V., Di Cosimo S., Reduzzi C., Pala V., Cappelletti V., Daidone M.G. (2017). How to Study and Overcome Tumor Heterogeneity with Circulating Biomarkers: The Breast Cancer Case. Semin. Cancer Biol..

